# A hybrid CNN-LSTM model for pre-miRNA classification

**DOI:** 10.1038/s41598-021-93656-0

**Published:** 2021-07-08

**Authors:** Abdulkadir Tasdelen, Baha Sen

**Affiliations:** 1 TOBB Technical Sciences Vocational School, Karabuk University, Karabuk, Turkey; 2 Department of Computer Engineering, Ankara Yıldırım Beyazıt University, Ankara, Turkey

**Keywords:** Computational biology and bioinformatics, Genetics, Engineering

## Abstract

miRNAs (or microRNAs) are small, endogenous, and noncoding RNAs construct of about 22 nucleotides. Cumulative evidence from biological experiments shows that miRNAs play a fundamental and important role in various biological processes. Therefore, the classification of miRNA is a critical problem in computational biology. Due to the short length of mature miRNAs, many researchers are working on precursor miRNAs (pre-miRNAs) with longer sequences and more structural features. Pre-miRNAs can be divided into two groups as mirtrons and canonical miRNAs in terms of biogenesis differences. Compared to mirtrons, canonical miRNAs are more conserved and easier to be identified. Many existing pre-miRNA classification methods rely on manual feature extraction. Moreover, these methods focus on either sequential structure or spatial structure of pre-miRNAs. To overcome the limitations of previous models, we propose a nucleotide-level hybrid deep learning method based on a CNN and LSTM network together. The prediction resulted in 0.943 (%95 CI ± 0.014) accuracy, 0.935 (%95 CI ± 0.016) sensitivity, 0.948 (%95 CI ± 0.029) specificity, 0.925 (%95 CI ± 0.016) F1 Score and 0.880 (%95 CI ± 0.028) Matthews Correlation Coefficient. When compared to the closest results, our proposed method revealed the best results for Acc., F1 Score, MCC. These were 2.51%, 1.00%, and 2.43% higher than the closest ones, respectively. The mean of sensitivity ranked first like Linear Discriminant Analysis. The results indicate that the hybrid CNN and LSTM networks can be employed to achieve better performance for pre-miRNA classification. In future work, we study on investigation of new classification models that deliver better performance in terms of all the evaluation criteria.

## Introduction

miRNAs (or microRNAs) are small, endogenous, and noncoding RNA constructs of about 22 nucleotides^1^. Cumulative evidence from biological experiments shows that miRNAs play a fundamental and important role in various biological processes such as regulation of gene expression by post-transcriptionally binding to 5'untranslated regions (UTR), coding sequences, or 3´UTR of target messenger RNAs (mRNAs)^2,3^. According to the latest release of an online miRNA database, miRBase (v22), there are 38,589 entries representing hairpin precursor miRNAs that express 48,860 mature miRNAs from 271 organisms such as humans, mice, rat, etc.^4^. The human genome, as a sub-category of the organism classification, contains 1917 annotated hairpin precursors, and 2654 mature sequences^4^. It is estimated that in mammals, approximately one-third of all protein-coding genes’ activities are controlled by miRNAs^5^. Several studies show that the deregulations of miRNAs are associated with many types of human diseases, e.g. cancer, cardiovascular diseases, or autoimmune diseases^6–27^. Due to these relationships between miRNAs and various diseases, studies to understand the functions, processes, and mechanisms of miRNAs are increasing dramatically^28^. Thus, how to classify miRNAs is a critical problem in computational biology.

The discovery of the first miRNA started in *Caenorhabditis elegans* in 1993 by Ambros and Ruvkun's studies. They found that the *lin-4* was a small noncoding RNA but not a protein-coding RNA^29–31^. Seven years later, in 2000, the second miRNA, *let-7*, was reported. Experimental results show that *let-7* consists of 21 nucleotide RNA and regulates timing in the transition from fourth level (L4) to adult *C. elegans’* larval development^32^.

**Figure 1:**
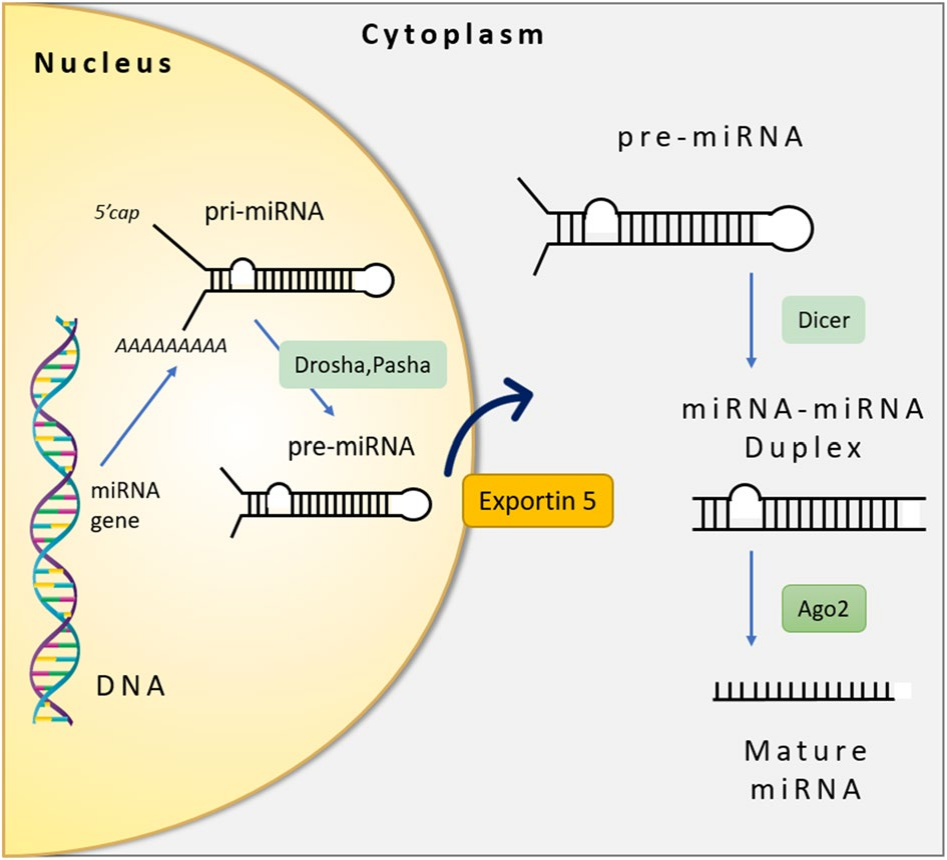
Overview of miRNA biogenesis.

The biogenesis of miRNAs involves several steps and cellular mechanisms (Fig. 1), some in the nucleus and some in the cytoplasm. Since those processes have some different pathways, pre-miRNAs can be categorized into two categories: mirtrons and canonical miRNAs. Compared to mirtrons, canonical miRNAs are more conserved and easier to be identified^33^. The first step of the biogenesis of miRNAs begins with the transcription of miRNA genes that make up primary miRNA hairpins called pri-miRNA^34–36^. In the canonical pathway, pre-miRNAs with the hairpin structure are formed by the microprocessor complex consisting of Drosha and DGCR by dividing pri-miRNAs in the nucleus^37,38^. Then, the pre-miRNAs are produced in the nucleus and transported into the cytoplasm by exportin-5. Following this, pre-miRNAs are cleaved in the cytoplasm into small RNA duplexes by another RNase III enzyme Dicer and finally, mature miRNA is produced^39,40^. In the mirtron pathway, for bypassing the nuclear enzyme Drosha, it uses splicing to produce short pre-miRNA hairpin introns^41,42^. The next steps of those pre-miRNAs are in the same pathway as canonical miRNAs^43^. Mirtrons can also be divided into three categories: canonical, 3′ tailed, and 5′ tailed due to their sequence and structure^42^. Compared to canonical miRNAs, mirtron hairpins and small RNAs have numerous distinguishing features^44,45^.

In previous studies, numerous computational methods, such as decision trees (DT), random forest (RF), and support vector machines (SVM), widely applied in miRNA identification and classification as applied in computational biology and healtcare^46–52^. Recently, Deep Learning (DL) methods are also frequently used to achieve better prediction accuracy compared with other traditional machine learning methods ^53–59^. The convolutional neural networks CNNs, a type of DL, have successfully employed for pre-miRNAs clasification^33,56^. For instance, Zheng et al.^33^ proposed a nucleotide-level CNN model. They encoded the sequences using “one-hot” encoding then padded each entry with the same shape. The model had convolutions and max-pooling layers. Their investigation showed that their CNN-based network feasible to apply to extract features from biological sequences. CNN-based methods outperform to identify the miRNAs and extract features automatically from the raw input data without detailed domain knowledge^60–63^. However, Park et al.^64^ show that the spatial information of these structures is as important as the structures that make up miRNAs. Therefore, they focused on only long-term dependencies and proposed an LSTM based framework to identify precursor miRNAs. Moreover, much research reveals that CNN-LSTM networks give a solution to use both structural characterization and spatial information together. A CNN-LSTM network is a combination of CNN layers for feature extraction on input data and LSTM layers to provide sequence prediction^65^. These networks are used in a variety of problems such as activity recognition, image description, video description, visual time series prediction, and generating textual annotations from image sequences^65,66^. Quang et al. proposed a hybrid CNN-LSTM framework^67^, DanQ, for predicting the function of DNA sequences. In this model, the convolution layer captures patterns, and the recurrent layer captures long-term dependencies. Similarly, Pan et al. proposed iDeepS^68^, to identify the binding sequence and structure patterns from RNA sequences. Their model extract features by using CNN and reveals possible long-term dependencies by using bi-directional LSTM (BLSTM). These successful studies show that utilizing both spatial and sequential features provides higher performance, especially in computational biology.

Many existing pre-miRNA classification methods focus on either sequential structure or spatial structure of pre-miRNAs. The main features that distinguish pre-miRNAs from each other are the types, number, and sequence order of amino acids that make up their fundamental structure. Hence, using a hybrid CNN-LSTM based network can give a solution to classify pre-miRNA facilitating with both spatial and sequential features of pre-miRNAs.

## Materials and methods

In this study, we presented a hybrid deep learning method for pre-miRNA classification based on both sequential and spatial structure of pre-miRNA by integrating two different networks respectively: CNN and LSTM. We first described the problem of pre-miRNA classification. Then, we introduced the dataset, which is used to train and evaluate the proposed method. The dataset consisted of human mirtrons and canonical miRNAs sequences^44^. For consistency, the same sequence data were used as the previous models^33,46^. CNN extracted features from the input data automatically. Thus, it gave a solution to the problem of manual extraction of features. LSTM layer was used to perform temporal modeling following the CNN layer that convolved the input data. Next, we gave comprehensive details about CNN, LSTM, and CNN-LSTM networks. Finally, we described our proposed method and how to implement it in detail. The method was implemented in python using the Keras library (2.4.3) https://github.com/keras-team/keras, with the backend of TensorFlow (2.4.0).

### Problem statement

Many existing pre-miRNA classification methods rely on manual future extraction. These methods focus on either spatial structure or sequential structure of pre-miRNAs. To overcome the limitations of previous models, we propose a nucleotide-level deep learning method based on a hybrid CNN and LSTM network together for pre-miRNAs classification. When we consider the structure and sequence of pre-miRNAs, it is clear that the problem is a binary sequence classification problem consisting of mirtrons and canonical miRNAs. In literature, several models including machine-learning methods have been developed to find a solution for the problematic classification. On the other hand, they have approximately 90% of accuracy. In this study, in the pre-miRNA classification, our goal was to show how to accurately predict classes with a hybrid CNN-LSTM network.

### Convolutional neural networks

A CNN network is a type of deep learning that produces excellent performance and has been widely applied to many applications such as image classification^69,70^, object detection^71,72^, speech recognition^73^, computer vision^74^, video analysis^75^, and bioinformatics^76,77^. Apart from the traditional neural networks, CNN includes numerous layers that make it deeper. Moreover, CNN uses weights, biases, and outputs via a nonlinear activation. A typical CNN architecture fundamentally consists of convolutional layers, pooling layers, and fully connected layers^63^.

The convolution operation used in the convolutional layer is as follows:

1 $$ F\left( {i,j} \right) = \left( {I*K} \right)\left( {i,j} \right) = \sum \sum I\left( {i + m,~j + n} \right)K\left( {m,n} \right)~ $$

where *I* for input matrix, *K* for a 2D filter of size m × n, and *F* for the output of a 2D feature map. And, the convolutional layer representation is with I*K.

### Long short-term memory networks

An LSTM network is a class of recurrent neural network (RNN) that uses memory blocks that assist to run successfully and learn faster than traditional RNN^78,79^. LSTM networks find practical solutions for the vanishing and exploding gradient problems of RNNs^80^. Apart from the RNNs, a cell state is used in the LSTM network to save long-term states including input, forget, and output gates. Thus, the network can remember previous data and connect it with the present ones. Also, it solves complicated tasks difficult to find a solution by previous RNNs^79,81^.

### CNN and LSTM networks

A CNN-LSTM model is a combination of CNN layers that extract the feature from input data and LSTMs layers to provide sequence prediction^65^. The CNN-LSTM is generally used for activity recognition, image labeling, and video labeling. Their common features are that they are developed for the application of visual time series prediction problems and generating textual annotations from image sequences^65,66^.

**Figure 2:**
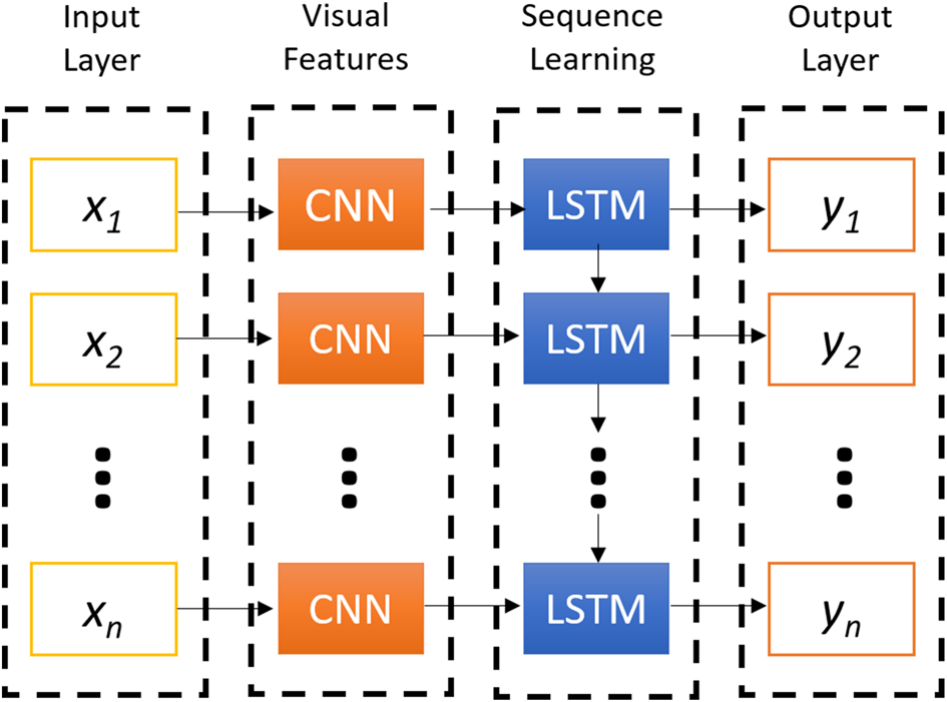
The basic architecture of the CNN-LSTM network.

Figure 2 shows the basic architecture of the CNN-LSTM network with the input layer, visual feature extraction, sequence learning, and output layer, respectively^65^.

### Training and test datasets

The dataset consists of mirtrons and canonical miRNAs’ data. We combined two different datasets in our preprocessing data phase with 707 (63%) canonical miRNAs and 417 (37%) mirtrons. The first dataset (Dataset 1) consisted of mirtrons and canonical miRNAs derived from miRBase (v21) according to the annotation of Wen et.al.^44^. Moreover, the second dataset (Dataset 2) was derived also from the study of Wen et al.^44^, which included 201 entries, putative mirtrons data. In total, we used 1,124 entries in our proposed model. The same dataset used to be consistent with Zheng et al. and Rorbach et al.^33,46^.

Stratified k-folds cross-validation (CV) is a resampling procedure that splits of the dataset into folds according to the output categories and ensures that each fold has the same proportion. It is useful for imbalanced datasets^82^. Hence, we used stratified 5-folds CV for training and evaluating our model^82^. At each iteration, it divided the data into training and test sets with a 80–20% split. In the next iteration, it used the other percentile as the training and test set.

Table 1 shows the distribution of the training and test datasets at each iteration in stratified 5-folds CV.

**Table 1:** Distribution of the training and test datasets in stratified 5-folds CV.

	Training dataset		Test dataset		Total	
	Sequence #	%	Sequence #	%	Sequence #	%
Mirtron	334	80	83	20	417	37
Canonical miRNA	566	80	141	20	707	63
Total	900	80	224	20	1124	100

### The preprocessing of the data

The entry with the most sequences had 164 bases. Therefore, we prepared each sequence of entries with the maximum length (164) by padding. The word "N" was used for keeping the sequences in the same length. Like Zheng et al.^33^, “one-hot” encoding is used to encode each base of the sequences (Table 2). Next, we converted each sequence into a vector with a dimension of (164, 4) by the vectorization process.

**Table 2:** “One-hot” encoding for the base sequence.

Base name	Encoded base			
A	1	0	0	0
U/T	0	1	0	0
G	0	0	1	0
C	0	0	0	1
N	0	0	0	0

### The method architecture

**Figure 3:**
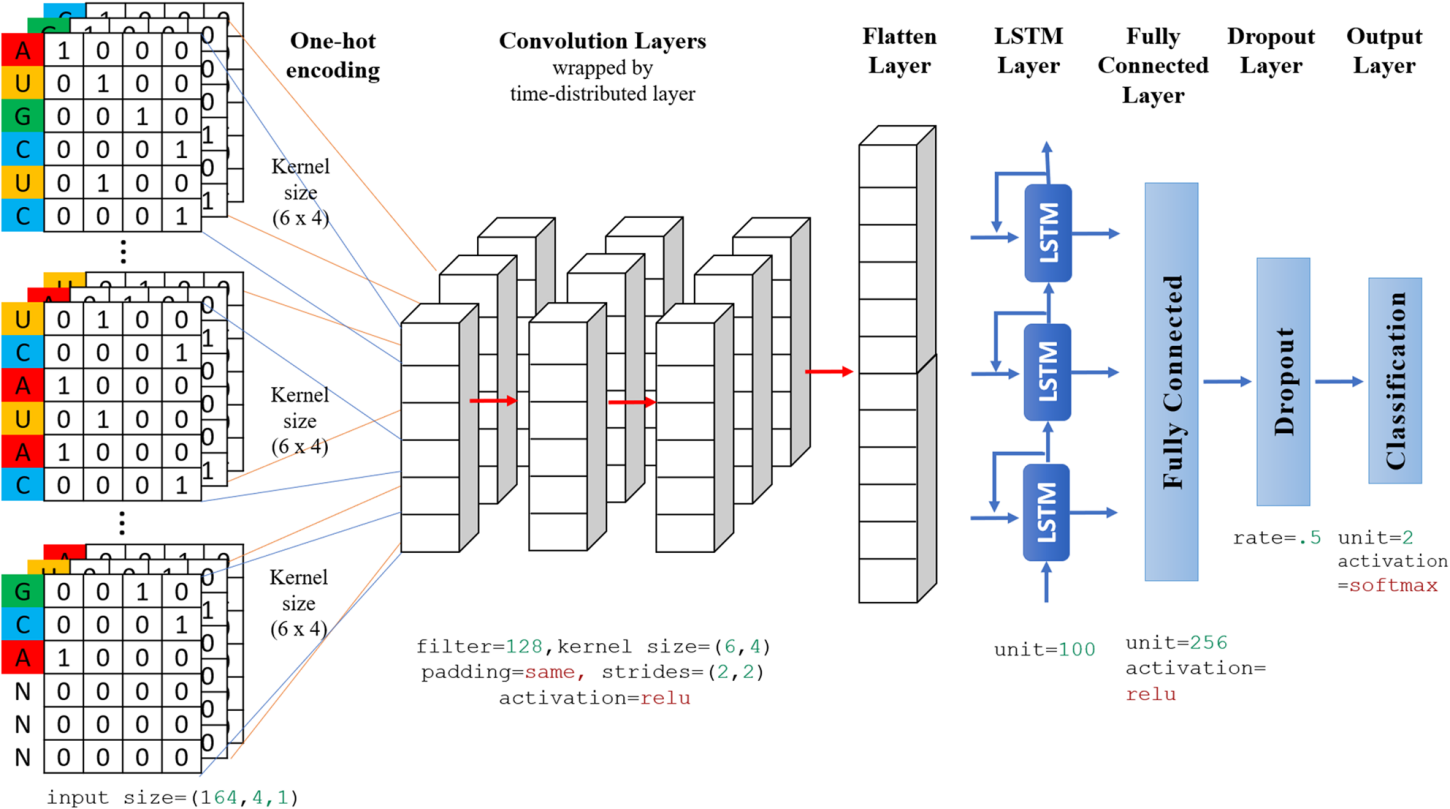
Detailed architecture with visualization of the proposed methodology.

We designed the architecture of our model with nine layers: an input layer, four CNN layers wrapped by the time-distributed layers, an LSTM layer, a dense layer, a dropout layer, and an output layer, respectively. Figure 3 shows the illustration of the architecture with visualization of our method. Before constructing the model, we ensured that each data has been transformed into an appropriate form to be used. In this case, we used the padding process to guarantee the length (which is 164) of each miRNA sequence similar by adding "N" for each blank. The next vectorization step was transforming the padded sequences to like a *m × n* matrix by using one-hot encoding.

When all data are padded and vectorized, the network became ready for the feature extraction process. In this stage, three convolution layers were used to automatically extract features from input sequences using the *relu* activation function. In these convolutional layers, 128 filters were used. The kernel's height was selected as 6 and the kernel's width was selected 4 for convolution operation. This kernel size gives higher performance^33^. In these convolutional stages. We wrapped the convolution layers in a time-distributed wrapper to reshape input data by adding extra dimension at the end. For concatenation of all extracted features, we employed a flatten layer for passing to the LSTM layer. Then, one LSTM layer was designed with 100 units following a dropout layer (0.5) on the fully connected layer. Finally, for binary classification, the *softmax* activation function was used for specifying outputs. The model was optimized for 30 epochs, 6 for batch size, and 0.1 for validation split by training. The validation dataset monitors the convergence in the training process so that the training of the model can be canceled early according to the change in this convergence. Besides, *adam* optimizer with a 0,001 learning rate for optimization and *categorical cross entropy* for loss function was preferred during the optimization process. *Adam* is one of the gradient descent algorithms that calculate adaptive learning rates for each momentum-like parameter^83^ and *categorical cross entropy* is one of the loss functions preferred when there are two or more one-hot encoded label classes^84^. It optimizes multi-class classification models with a *softmax* activation function.

Table 3 shows the model summary including the input layer, convolution layers, flatten layer, LSTM layer, fully-connected layer, softmax layer and classification layer with the shape and the number of the parameters.

**Table 3:** The method summary.

Layer name	Output shape	Param #
Input layer	(None, none, 164, 4, 1)	
Time distributed layer 1	(None, none, 82, 2, 128)	3200
Time distributed layer 2	(None, none, 42, 1, 128)	393344
Time distributed layer 3	(None, none, 21, 1, 128)	393344
Time distributed layer 4	(None, none, 2688)	0
LSTM layer	(None, 100)	1115600
Dense layer 1	(None, 256)	25856
Dropout	(None, 256)	0
Dense layer 2	(None, 2)	512

## Method evaluation

In this study, the evaluation of our method was measured on the test dataset. We calculated five different measurements for performance in the analysis: accuracy (Acc.), sensitivity (Sen.), specificity (Spe.), F1 score, and Matthews Correlation Coefficient (MCC). They are calculated for evaluating predictive capability with the number of true positives (TP), true negatives (TN), false positives (FP), and false negatives (FN) by the following equations:

Accuracy indicates the overall correctness of prediction:

2 $$ Acc = \frac{{TP~ + ~TN}}{{TP~ + ~FN + ~TN~ + ~FP~}} $$

Sensitivity, true positive rate, indicates the ratio of correctly classified actual positives:

3 $$ Sen = \frac{{TP}}{{TP + ~FN}} $$

Specificity, true negative rate, indicates the ratio of correctly classified actual negatives:

4 $$ Spe = \frac{{TN}}{{TN + ~FP}} $$

F1-Score is a combination of the precision and recall of the model by harmonic mean:

5 $$ F1~Score = \frac{{2TP}}{{2~TP + ~FP + FN}} $$

Matthews Correlation Coefficient (MCC) is a binary classifier that measures the quality:

6 $$ MCC = \frac{{TP \cdot ~~TN~{-}~FP \cdot ~~FN}}{{~\sqrt {\left( {{\text{TP~}} + {\text{~FP}}} \right)\left( {{\text{TP~}} + {\text{~FN}}} \right)~\left( {{\text{TN~}} + {\text{~FP}}} \right)\left( {{\text{TN~}} + {\text{~FN}}} \right)} }}. $$

## Results and discussion

Due to the automatic feature extraction without a comprehensive domain expert from pre-miRNAs sequences by using CNN and LSTM, we designed a hybrid method for the classification of pre-miRNAs. We started with preparing the dataset by converting the raw sequences to vectors using “one-hot” encoding. Next, all data were padded and vectorized. Then, we used three convolution layers to extract features automatically from input sequences using the *relu* activation function. For concatenation of all extracted features, we employed a flatten layer for passing to the LSTM layer. Then, one LSTM layer is designed with 100 units following a dropout layer (0.5) on the fully connected layer. Finally, for binary classification, the softmax activation function was used for the specifying outputs. Table 4 shows the performance results of our proposed network at each iteration. Additionally, we calculated mean, median, standard deviation, and confidence interval (CI) for each metric.

**Table 4:** Performance of the proposed CNN-LSTM network for each fold.

Fold #	Acc	Sen	Spe	F1 Score	MCC
1	0.942	**0.952**	0.937	0.924	0.878
2	**0**.**964**	0.916	**0**.**993**	**0**.**950**	**0**.**924**
3	0.933	0.952	0.922	0.914	0.862
4	0.929	0.929	0.929	0.907	0,850
5	0.946	0.928	0.957	0.928	0.885
Mean	**0**.**943**	**0**.**935**	**0**.**948**	**0**.**925**	**0**.**880**
Median	0.942	0.929	0.937	0.924	0.878
SD	0.014	0.016	0.029	0.016	0.028
95% CI	0.931–0.955	0.921–0.949	0.923–0.973	0.910–0.939	0.855–0.905

Table 5 shows the performance comparison of the average values of the proposed method with the previous methods. The prediction resulted in 0.943 (%95 CI ± 0.014) accuracy, 0.935 (%95 CI ± 0.016) sensitivity, 0.948 (%95 CI ± 0.029) specificity, 0.925 (%95 CI ± 0.016) F1 Score and 0.880 (%95 CI ± 0.028) MCC (Table 4) When compared to the closest results, our network revealed the best results for Acc., F1 Score, and MCC. These were 2.51%, 1.00%, and 2.43% higher than the closest result, respectively. The mean of sensitivity had the highest value like Linear Discriminant Analysis and ranked first. These ratios indicate that the hybrid CNN and LSTM networks can be employed to achieve better performance for pre-miRNA classification compared with previous methods. Even though the results show that our model has a higher ratio according to accuracy, sensitivity, F1 score, and MCC; we have a lower ratio (94.8%) of correctly classified true negatives. In imbalanced or skewed datasets, the number of examples of the minority class might not be sufficient for learning. As a result, the minority group is more often misclassified than the majority group^85,86^. The number of positive and negative samples in our training and test dataset is equally representative of the entire dataset. Thus, we solve the misclassification problem at the data preparation level.

**Table 5:** Performance comparison of pre-miRNA classification.

Method name	Acc	Sen	Spe	F1 Score	MCC
Proposed method*	**0.943**	**0.935**	0.948	**0.925**	**0.880**
CNN filter6 128^33^	0.920	0.871	0.970	0.916	0.845
CNN concat filters^33^	0.910	0.846	**0.975**	0.904	0.827
Support vector machines^46^	**	0.926	0.945	0.901	0.859
Random forest^46^	**	0.870	0.957	0.883	0.836
Linear discriminant analysis^46^	**	0.935	0.919	0.881	0.830
Logistic regression^46^	**	0.875	0.941	0.867	0.816
Decision tree^46^	**	0.861	0.943	0.863	0.808
Naive Bayes^46^	**	0.875	0.894	0.824	0.746

*Average value of the stratified 5-folds CV results.

**Data not available.

This study is an investigation of the pre-miRNA classification problem through a convolutional neural network and long short-term memory network. In contrast to other methods, we took into account both the sequence structure and the spatial information of each entry. The preprocessing of data is the first but the most important stage of our study. Indeed, inappropriate preparation of the data will cause the network to be trained incorrectly and will make it difficult to obtain reliable results. Thus, we checked all outputs after the encoding, padding, and vectorization process. In addition, cascading the different neural networks is another issue in the model construction. Inappropriate network design may increase the bias and cause unexpected results. Therefore, we ensured that all the layers cascaded correctly.

Hyper-parameters determine the general characteristics of deep neural networks. The number of the hidden units, order of the layers, batch size, optimizer selection, and learning rate, etc. directly affect the performance of the methods. In this study, we utilized the previous researcher's experiments in addition to our experiments. For instance, Zheng et al.^33^ discovered that kernel size (6 × 4) and unit number (128) of the CNN network produced the best results according to other sizes and numbers in the pre-miRNA classification. When we tested hyperparameters like Zheng et al.^33^, we obtained similar performance results as they did. In our future work, we will take into account the experiences we have gained in these studies and we will do more extensive hyper-parameter optimization to ensure performance increase.

Despite the promising performance of our model, there are still some limitations. The first limitation comes from the total number of entries (1124) in the datasets. Even though the datasets have well-defined data, it is important to feed the method with more training and testing data to obtain more reliable results. The second limitation is the unbalanced ratio of classes. In this study, the number of positive samples (417) was less than the number of negative samples (707). The ratio of positive and negative samples was approximately 1:1.7. This imbalanced ratio may lead to limit accuracy and other metrices. Thus, we will focus on more comprehensive datasets in the future research.

We consider that the quality and size of the related dataset are important for training a model and achieving robust classification prediction. In future studies, enhanced datasets may lead to the construction of more successful models in terms of similar evaluation parameters.

## Conclusion

In this paper, we proposed a nucleotide-level hybrid deep learning method based on a convolutional neural network and long-short term memory network together. In the data preprocessing phase, we used one-hot encoding to convert each base to a matrix of the same size by padding. Then, we employed three convolution layers wrapped by a time-distribution layer. For concatenation of all extracted features, we employed a flatten layer for passing to the LSTM layer. Then, we designed one LSTM layer following a dropout layer on the fully connected layer. Finally, for binary classification, the softmax activation function is used for specifying the outputs. Our results showed that the proposed method was successfully trained on the training dataset and had a better performance on the test dataset than the previous models.

The results indicated that the hybrid CNN and LSTM networks can be employed to achieve better performance for pre-miRNA classification. In future work, we will study on the investigation of new classification models that deliver better performance in terms of all the evaluation metrics.

## Supplementary Information

Supplementary Information 1.

Supplementary Information 2.

## References

[1] Hammond, S. M. An overview of microRNAs. *Adv. Drug Deliv. Rev.* **87** (n.d.). 10.1016/j.addr.2015.05.001 PMC4504744 25979468

[2] Bartel, D. P. MicroRNAs: genomics, biogenesis, mechanism, and function. *Cell*. **116**, 281–297 (n.d.). 10.1016/s0092-8674(04)00045-5 14744438

[3] Ambros, V. The functions of animal microRNAs. *Nature*. **431** (n.d.). 10.1038/nature02871 15372042

[4] Kozomara A, Birgaoanu M, Griffiths-Jones S (2019). MiRBase: from microRNA sequences to function. Nucleic Acids Res..

[5] Filipowicz W, Bhattacharyya SN, Sonenberg N (2008). Mechanisms of post-transcriptional regulation by microRNAs: are the answers in sight?. Nat. Rev. Genet..

[6] Meister, G. & Tuschl, T. Mechanisms of gene silencing by double-stranded RNA. *Nature*. **431** (n.d.). 10.1038/nature02873 15372041

[7] Karp, X. & Ambros, V. Encountering MicroRNAs in cell fate signaling. *Science (80-. )*. **310**, 1288–1289 (n.d.). 10.1126/science.1121566 16311325

[8] Garzon R, Calin GA, Croce CM (2009). MicroRNAs in cancer. Annu. Rev. Med..

[9] Zhao Y, Samal E, Srivastava D (2005). Serum response factor regulates a muscle-specific microRNA that targets Hand2 during cardiogenesis. Nature.

[10] Cheng Y, Zhang C (2010). MicroRNA-21 in cardiovascular disease. J. Cardiovasc. Transl. Res..

[11] Cheng Y, Ji R, Yue J, Yang J, Liu X, Chen H, Dean DB, Zhang C (2007). MicroRNAs are aberrantly expressed in hypertrophic heart: Do they play a pole in cardiac hypertrophy?. Am. J. Pathol..

[12] Sonkoly E, Wei T, Janson PCJ, Sääf A, Lundeberg L, Tengvall-Linder M, Norstedt G, Alenius H, Homey B, Scheynius A, Ståhle M, Pivarcsi A (2007). MicroRNAs: novel regulators involved in the pathogenesis of psoriasis?. PLoS ONE.

[13] Lee YS, Dutta A (2009). MicroRNAs in cancer. Annu. Rev. Pathol. Mech. Dis..

[14] Peng, Y. & Croce, C. M. The role of micrornas in human cancer. *Signal Trans. Target. Ther.* **15004** (n.d.). 10.1038/sigtrans.2015.4 PMC5661652 29263891

[15] Qin, S. & Zhang, C. Micrornas in vascular disease. *J. Cardiovasc. Pharmacol.* **57** (n.d.). 10.1097/FJC.0b013e318203759b PMC4517184 21052012

[16] Jamaluddin, M. S. Mirnas: roles and clinical applications in vascular disease. *Expert. Rev. Mol. Diagn*. **11** 79–89 (n.d.). 10.1586/erm.10.103 PMC3077058 21171923

[17] Dalal, S. R., Kwon, J. H. The role of microrna in inflammatory bowel disease. *Gastroenterol. Hepatol.* **6** (n.d.). PMC3033542 21437020

[18] Cheng, A. M., Byrom, M. W., Shelton, J., & Ford, L. P. Antisense inhibition of human miRNAs and indications for an involvement of miRNA in cell growth and apoptosis. *Nucleic Acids Res.* **33**, 1290–1297 (n.d.). 10.1093/nar/gki200 PMC552951 15741182

[19] Chapman, C. G. & Pekow, J. The emerging role of mirnas in inflammatory bowel disease: a review. *Ther. Adv. Gastroenterol*. **8**, 4–22 (n.d.). 10.1177/1756283X14547360 PMC4265084 25553076

[20] Hayes, J., Peruzzi, P. P., & Lawler, S. Micrornas in cancer: biomarkers, functions and therapy. *Trends Mol. Med.* **20**, 460–469 (n.d.). 10.1016/j.molmed.2014.06.005 25027972

[21] Kir, D., Schnettler, E., Modi, S., & Ramakrishnan, S. Regulation of angiogenesis by microRNAs in cardiovascular diseases. *Angiogenesis* (n.d.). 10.1007/s10456-018-9632-7. 10.1007/s10456-018-9632-7 29956018

[22] Lu J, Getz G, Miska EA, Alvarez-Saavedra E, Lamb J, Peck D, Sweet-Cordero A, Ebert BL, Mak RH, Ferrando AA, Downing JR, Jacks T, Horvitz HR, Golub TR (2005). MicroRNA expression profiles classify human cancers. Nature.

[23] Miska, E. A. How microRNAs control cell division, differentiation and death. *Curr. Opin. Genet. Dev.* **15** (n.d.). 10.1016/j.gde.2005.08.005 16099643

[24] Mandujano-Tinoco, E. A., Garcia-Venzor, A., Melendez-Zajgla, J., & Maldonado, V. New emerging roles of microRNAs in breast cancer. *Breast Cancer Res. Treat.* 10–1007 (n.d.). 10.1007/s10549-018-4850-7 29948402

[25] Singh, R. P. The role of miRNA in inflammation and autoimmunity. *Autoimmun. Rev.* **12**, 10–1016 (n.d.). 10.1016/j.autrev.2013.07.003 23860189

[26] Calin GA, Sevignani C, Dumitru CD, Hyslop T, Noch E, Yendamuri S, Shimizu M, Rattan S, Bullrich F, Negrini M, Croce CM (2004). Human microRNA genes are frequently located at fragile sites and genomic regions involved in cancers. Proc. Natl. Acad. Sci. USA..

[27] Li C, Feng Y, Coukos G, Zhang L (2009). Therapeutic microRNA strategies in human cancer. AAPS J..

[28] Alles J, Fehlmann T, Fischer U, Backes C, Galata V, Minet M, Hart M, Abu-Halima M, Grässer FA, Lenhof HP, Keller A, Meese E (2019). An estimate of the total number of true human miRNAs. Nucleic Acids Res..

[29] Lee, R. C., Feinbaum, R. L., & Ambros,V. The *C. elegans* heterochronic gene lin-4 encodes small RNAs with antisense complementarity to lin-14. *Cell*. (1993). 10.1016/0092-8674(93)90529-Y. 10.1016/0092-8674(93)90529-y 8252621

[30] Wightman, B., Ha, I., & Ruvkun, G. Posttranscriptional regulation of the heterochronic gene lin-14 by lin-4 mediates temporal pattern formation in *C. Elegans*. *Cell*. (1993). 10.1016/0092-8674(93)90530-4. 10.1016/0092-8674(93)90530-4 8252622

[31] Lee, R., Feinbaum, R., & Ambros, V. A short history of a short RNA. *Cell*. (2004). 10.1016/s0092-8674(04)00035-2. 10.1016/s0092-8674(04)00035-2 15055592

[32] Reinhart BJ, Slack FJ, Basson M, Pasquienelll AE, Bettlnger JC, Rougvle AE, Horvitz HR, Ruvkun G (2000). The 21-nucleotide let-7 RNA regulates developmental timing in *Caenorhabditis elegans*. Nature.

[33] Zheng X, Xu S, Zhang Y, Huang X (2019). Nucleotide-level convolutional neural networks for pre-miRNA classification. Sci. Rep..

[34] Siomi H, Siomi MC (2010). Posttranscriptional regulation of MicroRNA biogenesis in animals. Mol. Cell..

[35] Han, J. Molecular basis for the recognition of primary microRNAs by the Drosha-DGCR8 complex. *Cell*. **125,** 10–1016 (n.d.). 10.1016/j.cell.2006.03.043 16751099

[36] O’Brien J, Hayder H, Zayed Y, Peng C (2018). Overview of microRNA biogenesis, mechanisms of actions, and circulation. Front. Endocrinol. (Lausanne)..

[37] Denli, A. M., Tops, B. B., Plasterk, R. H., Ketting, R. F., & Hannon, G. J. Processing of primary micrornas by the microprocessor complex. *Nat*. **432** (n.d.). 10.1038/nature03049 15531879

[38] Gregory RI, Yan KP, Amuthan G, Chendrimada T, Doratotaj B, Cooch N, Shiekhattar R (2004). The microprocessor complex mediates the genesis of microRNAs. Nature.

[39] Lee Y, Ahn C, Han J, Choi H, Kim J, Yim J, Lee J, Provost P, Rådmark O, Kim S, Kim VN (2003). The nuclear RNase III Drosha initiates microRNA processing. Nature.

[40] Lund, E., Guttinger, S., Calado, A., Dahlberg, J. E., & Kutay, U. Nuclear export of microRNA precursors. *Science (80-. )*. **303**, 10–1126 (n.d.). 10.1126/science.1090599 14631048

[41] Ruby JG, Jan CH, Bartel DP (2007). Intronic microRNA precursors that bypass Drosha processing. Nature.

[42] Westholm JO, Lai EC (2011). Mirtrons: MicroRNA biogenesis via splicing. Biochimie.

[43] Berezikov E, Chung WJ, Willis J, Cuppen E, Lai EC (2007). Mammalian mirtron genes. Mol. Cell..

[44] Wen J, Ladewig E, Shenker S, Mohammed J, Lai EC (2015). Analysis of Nearly One Thousand Mammalian Mirtrons Reveals Novel Features of Dicer Substrates. PLoS Comput. Biol..

[45] Fromm, B. A uniform system for the annotation of vertebrate microRNA genes and the evolution of the human microRNAome. *Annu. Rev. Genet.* **49**, 213–242 (n.d.). 10.1146/annurev-genet-120213-092023 PMC4743252 26473382

[46] Rorbach, G., Unold, O., & Konopka, B. M. Distinguishing mirtrons from canonical miRNAs with data exploration and machine learning methods. *Sci. Rep.* **8**, 10–1038 (n.d.). 10.1038/s41598-018-25578-3 PMC5953923 29765080

[47] Gambhir S, Malik SK, Kumar Y (2016). Role of soft computing approaches in healthcare domain: a mini review. J. Med. Syst..

[48] Peker M, Şen B, Delen D (2015). Computer-aided diagnosis of Parkinson’s disease using complex-valued neural networks and mRMR feature selection algorithm. J. Healthc. Eng..

[49] Şen B, Peker M (2013). Novel approaches for automated epileptic diagnosis using FCBF selection and classification algorithms. Turk. J. Electr. Eng. Comput. Sci..

[50] Peker M, Sen B, Delen D (2016). A novel method for automated diagnosis of epilepsy using complex-valued classifiers. IEEE J. Biomed. Heal. Inform..

[51] Atasoy, F., Sen, B., Nar, F., & Bozkurt, I. Improvement of radial basis function ınterpolation performance on cranial ımplant design. *Int. J. Adv. Comput. Sci. Appl.*10.14569/ijacsa.2017.080811 (2017).

[52] Hofacker, I. L. Vienna RNA secondary structure server. *Nucleic Acids Res.* **31**, 3429–3431 (n.d.). 10.1093/nar/gkg599 PMC169005 12824340

[53] Ng KLS, Mishra SK (2007). De novo SVM classification of precursor microRNAs from genomic pseudo hairpins using global and intrinsic folding measures. Bioinformatics.

[54] Jiang P, Wu H, Wang W, Ma W, Sun X, Lu Z (2007). MiPred: Classification of real and pseudo microRNA precursors using random forest prediction model with combined features. Nucleic Acids Res..

[55] Sacar Demirci, M. D., Baumbach, J., Allmer, J. On the performance of pre-microRNA detection algorithms. *Nat. Commun.* **8**, 330. 10.1038/s41467-017-00403-z (n.d.). 10.1038/s41467-017-00403-z PMC5571158 28839141

[56] Zheng X, Fu X, Wang K, Wang M (2020). Deep neural networks for human microRNA precursor detection. BMC Bioinform.

[57] Do BT, Golkov V, Gürel GE, Cremers D (2018). Precursor microRNA identification using deep convolutional neural networks. BioRxiv..

[58] Cordero J, Menkovski V, Allmer J (2019). Detection of pre-microRNA with convolutional neural networks. BioRxiv..

[59] Xue,C. Classification of real and pseudo microrna precursors using local structure-sequence features and support vector machine. *BMC Bioinform*. **6** (n.d.). 10.1186/1471-2105-6-310 PMC1360673 16381612

[60] Huang DS (2004). A constructive approach for finding arbitrary roots of polynomials by neural networks. IEEE Trans. Neural Netw..

[61] Zhang Y, Zhang D, Mi G, Ma D, Li G, Guo Y, Li M, Zhu M (2012). Using ensemble methods to deal with imbalanced data in predicting protein-protein interactions. Comput. Biol. Chem..

[62] Albuquerque Vieira, J. P., & Moura, R. S. *An Analysis of Convolutional Neural Networks for Sentence Classification* (n.d.).

[63] Krizhevsky, A., Sutskever, I., & Hinton, G. E. ImageNet classification with deep convolutional neural networks. *Commun. ACM*. **60**, 10–1145 (n.d.).

[64] Park, S., Min, S., Choi, H., & Yoon, S. deepMiRGene: deep neural network based precursor microRNA prediction (2016). http://arxiv.org/abs/1605.00017. Accessed June 6, 2021.

[65] Donahue J, Hendricks LA, Rohrbach M, Venugopalan S, Guadarrama S, Saenko K, Darrell T (2017). Long-term recurrent convolutional networks for visual recognition and description. IEEE Trans. Pattern Anal. Mach. Intell..

[66] Sainath, T. N., Vinyals, O., Senior, A., & Sak, H. Convolutional, long short-term memory, fully connected deep neural networks. İn *ICASSP, IEEE International Conference on Acoustics, Speech, and Signal Processing—Proceedings*. 10.1109/ICASSP.2015.7178838(2015).

[67] Quang D, Xie X (2016). DanQ: A hybrid convolutional and recurrent deep neural network for quantifying the function of DNA sequences. Nucleic Acids Res..

[68] Pan X, Rijnbeek P, Yan J, BinShen H (2018). Prediction of RNA-protein sequence and structure binding preferences using deep convolutional and recurrent neural networks. BMC Genomics.

[69] Jia, Y., Shelhamer, E., Donahue, J., Karayev, S., Long, J., Girshick, R., Guadarrama, S., & Darrell, T. Caffe: convolutional architecture for fast feature embedding. İn *MM 2014, Proceedings of 2014 ACM Conference on Multimedia*. 10.1145/2647868.2654889 (2014).

[70] Zhao, Z. Q., Xie, B. J., Cheung, Y. M., & Wu, X. Plant leaf identification via a growing convolution neural network with progressive sample learning. İn *Lecture Notes Computer Science (Including Subser. Lecture Notes Artificial Intellgent Lecture Notes Bioinformatics)* (2015). 10.1007/978-3-319-16808-1_24.

[71] Xiang, Y., Choi, W., Lin, Y., & Savarese, S. Subcategory-aware convolutional neural networks for object proposals and detection. İn *Proceedings of 2017 IEEE Winter Conference on Application Computer Vision, WACV 2017* (2017). 10.1109/WACV.2017.108.

[72] Galvez, R. L., Bandala, A. A., Dadios, E. P., Vicerra, R. R. P., & Maningo, J. M. Z. Object detection using convolutional neural networks. İn *IEEE Reg. 10 Annual Interational Conference Proceedings/TENCON* (2019). 10.1109/TENCON.2018.8650517.

[73] Abdel-Hamid, O., Mohamed, A. R., Jiang, H., & Penn, G. Applying convolutional neural networks concepts to hybrid NN-HMM model for speech recognition. İn *ICASSP, IEEE International Conference on Acoustics, Speech, and Signal Processing*.: Proceedings (2012). 10.1109/ICASSP.2012.6288864.

[74] Karpathy, A., Toderici, G., Shetty, S., Leung, T., Sukthankar, R., & Li, F. F. Large-scale video classification with convolutional neural networks. İn *Proceedings of IEEE Computer Society Conference on Computer Vision and Pattern Recognition* (2014). 10.1109/CVPR.2014.223.

[75] Ngiam, J., Khosla, A., Kim, M., Nam, J., Lee, H., & Ng, A. Y. Multimodal deep learning. İn *Proceedings 28th Internationl Conference on Machanical Learning ICML 2011* (2011).

[76] Alipanahi B, Delong A, Weirauch MT, Frey BJ (2015). Predicting the sequence specificities of DNA- and RNA-binding proteins by deep learning. Nat. Biotechnol..

[77] Zhou J, Troyanskaya OG (2015). Predicting effects of noncoding variants with deep learning-based sequence model. Nat. Methods..

[78] Gers, F. Long short-term memory in recurrent neural networks. *Neural Comput.* (2001).

[79] Hochreiter S, Schmidhuber J (1997). Long short-term memory. Neural Comput..

[80] Hochreiter, S. The vanishing gradient problem during learning recurrent neural nets and problem solutions. *Int. J. Uncertain. Fuzz. Knowl. Based Syst.*10.1142/S0218488598000094 (1998).

[81] Greff K, Srivastava RK, Koutnik J, Steunebrink BR, Schmidhuber J (2017). LSTM: a search space odyssey. IEEE Trans. Neural Netw. Learn. Syst..

[82] He, H., & Ma, Y. Imbalanced learning: foundations, algorithms, and applications (2013).

[83] Kingma, P. E. & Ba, J. L. Adam: a method for stochastic optimization. İn *3rd The International Conference on Learning Representations ICLR 2015—Conference on Tracking Proceedings, International Conference on Learning Representations, ICLR* (2015). https://arxiv.org/abs/1412.6980v9. Accessed June 7, 2021.

[84] Probabilistic losses (n.d.). https://keras.io/api/losses/probabilistic_losses/#categoricalcrossentropy-class. Accessed June 7, 2021.

[85] Johnson JM, Khoshgoftaar TM (2019). Survey on deep learning with class imbalance. J. Big Data..

[86] Kotsiantis, S., Kanellopoulos, D., Pintelas, P. E., Kanellopoulos, D., & Pintelas, P. Handling imbalanced datasets: a review data preprocessing view project machine learning and data mining view project handling imbalanced datasets: a review (n.d.). https://www.researchgate.net/publication/228084509. Accessed June 8, 2021.

